# Effect and Neuroimaging Mechanism of Electroacupuncture for Vascular Cognitive Impairment No Dementia: Study Protocol for a Randomized, Assessor-Blind, Controlled Clinical Trial

**DOI:** 10.1155/2020/7190495

**Published:** 2020-02-26

**Authors:** Ruizhu Lin, Jia Huang, Jianfeng Xu, Jing Tao, Ying Xu, Jiao Liu, Weilin Liu, Shengxiang Liang, Minguang Yang, Lidian Chen

**Affiliations:** ^1^Rehabilitation Medicine College, Fujian University of Traditional Chinese Medicine, Fuzhou 350122, China; ^2^General Hospital of Ningxia Medical University, Yinchuan, Ningxia, China; ^3^Fujian University of Traditional Chinese Medicine, Fuzhou 350122, China

## Abstract

Vascular cognitive impairment no dementia (VCIND) is likely to develop into vascular dementia (VD) without intervention. The clinical efficacy of electroacupuncture (EA) for VCIND has been previously demonstrated. However, the neuroimaging mechanism of EA for VCIND has not been elucidated clearly. This trial is designed to provide solid evidence for the efficacy and neuroimaging mechanism of EA treatment for patients with VCIND. This ongoing study is an assessor-blind, parallel-group, randomized controlled trial. 140 eligible subjects will be recruited from the General Hospital of Ningxia Medical University and randomized into either the electroacupuncture (EA) group or the control group (CG). All subjects will receive basic treatment, and participants in the CG will receive health education performed weekly. Except for basic treatment and health education, participants in the EA group will receive treatment 5 times per week for a total of 40 sessions over 8 weeks. The primary outcome in this study is Montreal Cognitive Assessment (MoCA), and the secondary outcomes are Auditory Verbal Learning Test (AVLT), Stroop color-naming condition (STROOP), Rey–Osterrieth Complex Graphics Testing, and resting-state functional magnetic resonance imaging (rs-fMRI). All of the outcome measures will be assessed at baseline and 8 weeks of intervention. The medical abstraction of adverse events will be done at each visit. The results of this trial will demonstrate the efficacy and neuroimaging mechanism of EA treatment for VCIND, thus supporting EA treatment as an ideal choice for VCIND treatment. The trial was registered at the Chinese Clinical Trial Registry on 28 July 2018 (ChiCTR1800017398).

## 1. Introduction

Vascular cognitive impairment (VCI) is cognitive dysfunction caused by cerebrovascular disease or factor, ranging from mild cognitive dysfunction (vascular cognitive impairment no dementia, VCIND) to overt dementia (vascular dementia, VD) [1]. Therefore, VCIND constitutes an intermediate stage between normal aging and vascular dementia [2]. VCIND manifests mainly cognitive deficits in at least one cognitive domain without impairment of activities of daily living (ADL) [3, 4]. A widely shared view is that up to 46% of patients with VCIND progress to VD after 5 years of diagnosis are without treatment [5]. Therefore, the early intervention of VCI, particularly VCIND, which would greatly reduce the chances of developing VD in later life, is particularly important.

In fact, previous research on EA has demonstrated that EA is an effective treatment to improve cognitive function by VCIND [6, 7]. One study revealed that, after 8 weeks of scalp EA treatment, the Mini-Mental State Examination score increased from 25.1 ± 0.9 to 28.4 ± 1.4, and the picture recognition increased from 10.1 ± 3.1 to 12.5 ± 3.1. Therefore, opposed to conventional treatment, EA is a promising and simple method of improving the cognitive function of the cognitive impairment patients [8]. In an animal model of cognition disorder, it has been supported that EA can improve memory and reduce neuroinflammation associated with dementia, which means to have neuroprotective effects for the brain [9]. However, the mechanism of EA in the treatment of VCIND remains unclear, especially in neuroimaging. Currently, rs-fMRI has been recognized as one of the most promising techniques for neuroscientists to decode brain activity, especially for elderly or patients with cognitive impairment [10, 11]. Neuroimaging studies have verified that the human brain has a default functional network (DMN) at rest [12]. Previous studies have demonstrated that cognitive disorders in subcortical vascular cognitive impairment (SVCI) are associated with alteration in resting DMN [13].

On the other hand, many studies have indicated that acupuncture regulates the activity of some cortical and subcortical brain regions, including the alteration of DMN [14, 15]. Therefore, we hypothesize that EA may improve the cognitive function of VCIND due to changes with DMN. Through this proposed randomized controlled trial (RCT), we plan to explore the potential mechanism of EA in improving cognitive function in patients with VCIND.

Therefore, during this trial, our main aim is to assess the effect of EA on cognition function for VCIND. Our second aim will be to explore the neuroimaging mechanism after EA treatment on VCIND.

## 2. Methods

### 2.1. Study Design and Setting

**Figure 1:**
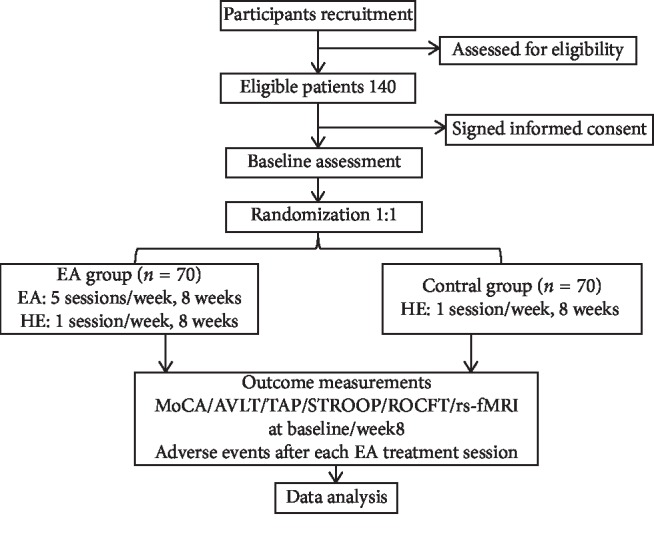
The planned flowchart of the trail. EA: electroacupuncture; HE: health education; MOCA: Montreal Cognitive Assessment; AVLT: Auditory Verbal Learning Test; TAP: Test of Attentional Performance; STROOP: Stroop color-naming condition; ROCFT: Rey–Osterrieth Complex Graphics Testing; rs-fMRI: resting-state functional MRI.

This study is an assessor-blind parallel-group RCT to explore the clinical efficacy and neuroimaging mechanism of EA for improving the cognitive function of VCIND patients. It will be performed in the General Hospital of Ningxia Medical University. A total of 140 eligible subjects will be randomly divided into 2 groups, namely, (1) EA group and (2) CG group, in a ratio of 1 : 1. All subjects will receive basic treatment with essential drugs, including hypotension, hypoglycemics, and antiplatelet and hypolipidemic drugs. In the CG group, they will only receive health education once a week, a total of 8 times. Except for basic treatment and health education, participants in the EA group will receive EA treatment that will be performed for 30 min per day and 5 days per week for 8 weeks. Operators with more than 5 years of clinical experience will perform the interventions. The 8-week intervention will be conducted. Outcome measurements include Montreal Cognitive Assessment (MoCA), Auditory Verbal Learning Test (AVLT), Test of Attentional Performance (TAP), Stroop color-naming condition (STROOP), Rey–Osterrieth Complex Graphics Testing (ROCFT), resting-state functional MRI (rs-fMRI), and adverse events. The planned flowchart for this trial is shown in Figure 1. The time point of assessment is provided in Table 1. Ethical approval has been provided with the Ethics Committee of the General Hospital of Ningxia Medical University in April 2018 (Additional file 1). Besides, General Hospital of Ningxia Medical University is also the project undertaking unit, which is responsible for coordinating the activities of all departments (e.g., trial registries, researcher training, informed consent of the participants, and data management). If there is an important protocol to be modified, it will notify the relevant parties to hold a coordination meeting. This protocol follows Standard Protocol Items: Recommendations for Interventional Trials (SPIRIT) (see Additional [Supplementary-material supplementary-material-1] in Supplementary Materials) [16].

### 2.2. Sample Size Calculation

In this procedure, the total score of the Montreal Cognitive Assessment Scale was invoked as the main effect index. The effect value was 0.9 in the treatment group and 0.725 in the control group [17], 0.8 in power and 0.05 in alpha. The sample size was estimated at PASS 24.0 software to be 116 cases, i.e., 58 cases in each group. Allowing 20% dropout rate, the total sample size was expected to be 140 patients (70 in each group).

### 2.3. Participants and Recruitment

A total of 140 right-handed participants will be recruited from community VCIND patients in Yinchuan, Ningxia Hui Autonomous Region, China. Eligible patients must comply with the inclusion and exclusion criteria. Participants were recruited through multimodal strategies, including print advertisements, WeChat, volunteer recommendation, and community publicity. All participants will undergo a baseline evaluation, including complete sociodemographic and clinical data collection. Meanwhile, all participants will be invited to voluntarily sign the informed consent.

### 2.4. Diagnostic Criteria

Compiling Guidelines for Diagnosis and Treatment of Vascular Cognitive Impairment with Reference to the Neurological Society of Chinese Medical Association [18].

(1) Cognitive impairment: the main complaint or informed person-reported cognitive impairment, and objective examination also had evidence of cognitive impairment, and/or objective examination confirmed that cognitive function was lower than before. (2) Vascular factors: including vascular risk factors, history of stroke, focal signs of nervous system, and evidence of cerebrovascular disease shown by imaging, not necessarily all of the above. (3) There is a causal relationship between cognitive impairment and vascular factors. (4) The daily ability is basically normal, but the complex instrumental daily ability can have slight damage. (5) It does not meet the diagnostic criteria of dementia.

### 2.5. Inclusion Criteria

Patients aged 50–75 years who (1) meet the diagnostic criteria of VCIND described above; (2) MoCA score of 18–26 [19, 20]; (3) are right-handed; (4) elapsed time after stroke ≥6 months; (5) have no acupuncture treatment in the last six months; and (6) volunteer to participate in the research and sign the informed consent were included in the study.

### 2.6. Exclusion Criteria

Patients who (1) have awareness disorders, severe visual, hearing, and aphasia, and other health assessment failures; (2) have memory and executive dysfunction caused by vascular dementia, Alzheimer's disease, hypothyroidism, brain trauma, etc.; (3) have comorbidity including tumors and severe heart, liver, kidney, hematopoietic system, and endocrine system diseases; (4) have psychiatric history, such as personality disorders and schizophrenia; and (5) have cognitive impairment caused by depression, Baker Depression Scale (>10 points) will be excluded from the study.

### 2.7. Dropout Criteria

Patients who (1) have serious adverse events, which are not advisable to continue the experiment; (2) have a secondary stroke after enrollment; and (3) quit the RCT voluntarily will be considered as having dropped out.

### 2.8. Randomization and Allocation

After meeting the selection criteria, 140 eligible participants will be randomly assigned to the EA group and the CG group in a 1 : 1 allocation ratio. The random grouping sequence will be performed according to a random list of numbers generated by the randomization center of Ningxia Medical University and hidden by the opaque and closed envelope. The random sequence will be managed by specific personnel who contact no participant and are not involved in the data collection or analysis.

All participants will receive the following free services, including assessment, functional magnetic resonance imaging (both EA group and CG group), health education (both EA group and CG group), and EA treatment (only EA group) and should adhere to all assessment and treatment schedules as much as possible.

### 2.9. Blinding

A single-blind method will be used. Outcome assessors, data manager, and statistics analyzer will be blinded to group allocation. However, participants will not be blinded because each participant knows his or her group. Therefore, the results of the assessment will be kept confidential to the participants to improve the accuracy of the test indicators and assure the objectivity and reliability of the evaluation. Blinding will be maintained during the whole study process. After completion of the statistical analysis, the blind code will be disclosed.

## 3. Treatment

### 3.1. Basic Treatment

Based on their current medical history, all participants received routine treatment with essential drugs, including hypotension, hypoglycemia, antiplatelet aggregation, and lipid regulation.

### 3.2. Interventions

All participants (2 groups) will be given health education once a week understanding the management of risk factors such as hypertension, diabetes, hyperlipidemia, and obesity and the drug management of these diseases. In addition, participants in the EA group will receive electroacupuncture treatment five sessions a week. Participants will be intervened in screen-separated personal space and will not be allowed to communicate with each other. Treatment will be conducted within 8 consecutive weeks.

### 3.3. Control Group

The CG group will only receive health education without electroacupuncture treatment. After finishing this trial, electroacupuncture treatment can be freely provided if they need, which is the same as the EA group.

### 3.4. Electroacupuncture Group

Electroacupuncture treatment details are presented in Table 2 and are based on the revised Standards for Reporting Interventions in Clinical Trials of Acupuncture (STRICTA) 2010 checklist [24]. In the sitting position, wipe Baihui and Shenting with alcohol, then obliquely pierce the steel needle (Suzhou Medical Supplies Factory Co., Ltd., Jiangsu, China) with the 0.3 ∗ 25 mm into the skin of the head 0.5 inches deep, and then perform appropriate operations at each point (including lifting, thrusting, and rotating) for 10 seconds to achieve the needling sensation (De qi sensation). Finally, these two needles will be connected to a SDZ-II Huatuo Electroacupuncture Instrument (Hengshui Hengkang Medical Equipment Co., Ltd., Hebei, China) with electrode clamps for 30 minutes per day, 5 days per week, 8 consecutive weeks.

### 3.5. Outcome Assessment

All outcomes will be measured by several experienced assessors who have been trained to administer these assessments and are blinded to the randomization group after the baseline visit for evaluation. Meanwhile, ensure that each subject's pre- and postevaluations are conducted by the same assessor.

### 3.6. Primary Outcome

The primary outcome of this study is the changes in the score on MoCA from baseline to 8 weeks after intervention between the two groups and within groups. It is widely used for cognitive function assessment and can reflect the whole cognitive level of individuals. The MoCA has a maximum score of 30 points divided into eight parts, namely, visual space/executive ability (5 points), name (3 points), memory (5 points), attention (3 points), computing (3 points), language fluency (3 points), abstraction (2 points), and orientation (6 points). MoCA has excellent reliability and validity and is sensitive enough for clinical and research practice [25].

### 3.7. Secondary Outcomes

Secondary outcomes include Auditory Verbal Learning Test (AVLT), Test of Attentional Performance (TAP), Stroop color-naming condition (STROOP), Rey–Osterrieth Complex Graphics Testing (ROCFT), and rs-fMRI, all of which will be evaluated at the same time points as the assessment of the primary outcome.

In this study, the AVLT will be used to evaluate language learning and memory function, which include three tests: immediate recall, short-term delayed recall, and long-term delayed recall [26].

Attention testing is accomplished by the TAP 2.3 attention testing system of PSYTEST Company to test attention span, duration, and functions mainly, which includes four test items: alertness, transfer attention, distracting attention, and response/nonresponse [27].

The STROOP compiled by prime psychological programming software consists of three parts: neutral test, consistency test, and reverse test. It mainly tests the executive function [28].

The visuospatial function was tested by ROCFT, which included the assessment of visual-spatial structure and visual memory [29].

Rs-fMRI will be performed using Philips Ingenia 3.0 T magnetic resonance imager. The data will be scanned by the Department of Radiology, General Hospital of Ningxia Medical University. The specific operation scheme is as follows.

### 3.8. Brain MRI Protocol

For participants, the imaging data which include conventional brain T1-weighted image (T1WI) and resting fMRI will be collected and acquired using a 3.0 T Ingenia scanner (Philips, the Netherlands) at the General Hospital of Ningxia Medical University. T1-weighted sequence and resting fMRI are shown as the following parameters, respectively [21].

T1: repetition time (TR) = 8.1 ms, echo time (TE) = 3.7 ms, flip angle (FA) = 8°, number of slices = 150, slice thickness = 1 mm, voxel size = 1 × 1 × 1 mm^3^, FOV = 240 mm ×240 mm, and matrix = 240 × 222.

Resting fMRI: TR = 2000 ms, TE = 30 ms, flip angle = 90°, number of slices = 36, slice thickness = 3.8 mm, voxel size = 3.01 × 3.09 × 3.80 mm^3^, FOV = 241 mm × 241 mm, matrix = 80 × 78, and phases per location = 240.

Participants will be requested to lie quietly in the scanner and close their eyes without thinking during the data acquisition process. Meanwhile, earplugs will be utilized to reduce the noise when the machine is running. Each scan lasted 1380 seconds.

### 3.9. Data Collection, Management, and Monitoring

Well-designed electronic CRF tables will be used for data collection. During the data collection process, a dependent research assistant (RA) will responsible for quality control. Two independent researchers will check the Case Report Form and Adverse Events Form carefully and input them into the computer to ensure data accuracy.

The project manager will have access to the data and be responsible for the initial data cleansing, identification, coding, and conversion to the appropriate data analysis format. All the original forms will be archived in the clinical research center of the General Hospital of Ningxia Medical University. Then, all data are treated with the utmost confidentiality and made anonymous to anyone outside the study.

The Data and Safety Monitoring Board (DSMB) of the clinical evaluation center of the General Hospital of Ningxia Medical University are responsible for data monitoring, i.e., obtaining any interim results and making the final decision to terminate the trial if any serious acupuncture-related EA occurs.

### 3.10. Statistical Analysis

IBM SPSS Statistics 24.0 software will be used to conduct the whole data analysis by an independent statistician who is unaware of group assignments. All continuous variables will be presented as mean ± standard deviation (SD), and categorical variables will be presented as numbers (%). The statistical significance level is considered to be 95% CIs (two-sided alpha *P* < 0.05).

The effect of the intervention will be assessed as the change in outcome measures between the baseline and eight weeks (after treatment sessions end), using a repeated-measures analysis of variance (ANOVA) and chi-square test.

The difference in effect between the CG group and the EA group will be assessed by comparing changes in the MoCA score and secondary outcomes including language learning and memory function, attention function, executive function, and visuospatial function using the independent *t*-test or Mann–Whitney *U* test on continuous variables and chi-square test or Fisher's exact test on categorical variables.

Intention-to-treat analyses will be used for all outcomes. A multiple imputation method will be carried out to fill in missing data for outcomes.

The structure and function of functional magnetic resonance will be analyzed by SPM software based on the MATLAB platform. Neural network analysis techniques such as VBM, ROI, and ICA will be used to compare the differences in functional connectivity changes between groups by independent sample *t*-test. FDR correction will be used for multiple comparison and correction.

### 3.11. Quality Control

To ensure the quality of this trial, the DSMB of the clinical evaluation center of the General Hospital of Ningxia Medical University will monitor regularly and strictly, consisting of informed consent, participant screening, intervention, serious EA records, statistical analysis, and data management.

### 3.12. Investigation of Adverse Events and Safety

During the acupuncture treatment, subjects will be informed about adverse events such as local bleeding, subcutaneous hematoma, or pain associated and serious adverse events such as dizziness and palpitation in advance. Any adverse events and serious adverse events during treatment will be reported in the Case Report Form (CRF) and investigated, including its type and frequency, and appropriate treatments for the associated symptoms will be provided immediately to minimize serious adverse events. If a serious adverse event occurs, the case will be reported to the Data and Safety Monitoring Board (DSMB) of the clinical evaluation center of the General Hospital of Ningxia Medical University immediately and the blinding code will be broken. If necessary, even part or all of the trial will be suspended until further instructions are expected to be available. We will pay for the treatment of the subjects with serious adverse events until recovery.

### 3.13. Trial Status

The project is ongoing recruitment and intervention.

## 4. Discussion

Governor vessel is one of the important meridians of the human body, which has the function of Governor Yang Qi and regulating spirit. Baihui and Shenting are very important acupoints on the Governor vessel. Baihui is the place where Yang Qi of the Governor vessel at its peak and Shenting is the place where wisdom is concentrated. Combining the stimulation of the two acupoints can strengthen the function of regulating the Du meridian and the spirit. Considering the importance of Du Meridian, Baihui and Shenting acupoints will be mainly selected in the treatment of cognitive disorders in traditional Chinese medicine, based on previous studies [22] and a large number of literature [23, 30].

On the other hand, fewer serious adverse events indicate that acupuncture is one of the safest nonpharmaceutical treatments [31, 32]. Although the clinical effectiveness of EA in improving cognitive impairment and/or major symptoms of dementia has been established, the central mechanism of EA is not clear yet. Neuroimaging, as an effective means to explore the central mechanism of acupuncture's effects, is widely used in acupuncture research [33]. Resting-state functional connectivity of the brain network reflects the inherent and spontaneous neural activity of the brain activity pattern; it is one of the most important technologies for assessing brain function and has good clinical applicability.

Based on the previous literature, acupuncture could increase DMN connectivity with pain-, affective-, and memory-related brain areas [34, 35], but evidence in vascular cognitive impairment is lacking. For this reason, we chose EA to intervene in VCIND, to explore its clinical efficacy and central mechanism, especially the changes of brain DMN.

Some limitations of this study should be noted. First, this trial is only one RCT without follow-up; whether these patients will convert to VD in the future is uncertain. Therefore, a follow-up to track the dynamic evolution of these patients is planned. Second, there is no therapeutic comparison in our study. We only set up a blank comparison group for observation. Therefore, we plan to establish a sham acupuncture group or drug group in future studies. Third, this trial is also restricted to a single center.

In conclusion, the results of this study following the CONSORT [36] and STRICTA [24] guidelines are expected to elucidate the clinical effects and neuroimaging mechanism of EA for VCIND, providing more solid evidence for traditional Chinese medicine (TCM) practitioners to give EA treatment for VCIND. Besides, we hope that our further research study will be improved and carried out in multicentered hospitals with an expanded sample size according to these experiences.

## Figures and Tables

**Table 1:** The study schedule for enrollment, interventions, and assessments.

Items	Before enrollment −2 to −1 week	Intervention period 1–8 weeks	Outcome assessment 9 weeks
Inclusion criteria	×		
Exclusion criteria	×		
Informed consent	×		
Baseline	×		
Randomization and allocation	×		
Intervention		×	
Baker Depression Scale test	×		
MoCA	×		×
AVLT	×		×
TAP	×		×
STROOP	×		×
ROCFT	×		×
rs-fMRI	×		×
Adverse events		×	
Reasons of dropout and withdrawals		×	

**Table 2:** STRICTA checklist (details of intervention).

	Item	Detail
1. Acupuncture rationale	(1a) Style of acupuncture	Electroacupuncture based on traditional Chinese medicine theory
	(1b) Reasoning for treatment provided, based on historical context, literature sources, and/or consensus methods, with references where appropriate	Acupuncture points to be used in this study were Baihui (GV20) and Shenting (GV24) located at the head. Based on previous studies [21] and a large number of literature [22, 23], Baihui (GV20) and Shenting (GV24) will be mainly selected as acupuncture points for VCI treatment.
	(1c) Extent to which treatment was varied	Only EA group will receive electroacupuncture treatment

2. Details of needling	(2a) Number of needle insertions per subject per session	2
	(2b) Names of points used	Baihui (GV20) and Shenting (GV24)
	(2c) Depth of insertion, based on a specified unit of measurement	0.5 inches deep
	(2d) Response sought	“De qi” sensation
	(2e) Needle stimulation	Electroacupuncture
	(2f) Needle retention time	30 minutes
	(2g) Needle type	Sterilized stainless steel needle with the 0.3 ∗ 25 mm (Suzhou Medical Supplies Factory Co., Ltd., Jiangsu, China)

3. Treatment regimen	(3a) Number of treatment sessions	40 sessions (8 consecutive weeks)
	(3b) Frequency and duration of treatment sessions	30 minutes per day, 5 days per week

4. Other components of treatment	(4a) Details of other interventions administered to the acupuncture group	(a)All participants received routine treatment with essential drugs(b)All participants will be given health education once a week
	(4b) Setting and context of treatment, including instructions to practitioners and information and explanations to patients	The study will be conducted in the General Hospital of Ningxia Medical University, and all information will be provided to the subjects

5. Practitioner background	(5) Description of participating acupuncturists	A medicine acupuncture after completing 5 years of Chinese medicine undergraduate course who has more than 5 years of clinical experience

6. Control or comparator interventions	(6a) Rationale for the control or comparator in the context of the research question, with sources that justify this choice	The control group was not treated with sham acupuncture or placebo acupuncture
	(6b) Precise description of the control or comparator. If sham acupuncture or any other type of acupuncture-like control is used, provide details as for items 1 to 3 above	None

STRICTA: Standards for Reporting Interventions in Clinical Trials of Acupuncture. EA: electroacupuncture.

## Data Availability

Data and materials are available upon request from the corresponding author.

## Abbreviations

VCIND: Vascular cognitive impairment no dementia
EA: Electroacupuncture
VD: Vascular dementia
CG: Control group
MoCA: Montreal Cognitive Assessment
AVLT: Auditory Verbal Learning Test
STROOP: Stroop color-naming condition
ROCFT: Rey–Osterrieth Complex Graphics Testing
rs-fMRI: Resting-state functional magnetic resonance imaging.

## Additional Points

Recruitment began in August 2018 (Protocol version 1.0).

## Supplementary Materials

Supplementary Materials SPIRIT 2013 checklist: recommended items to address in a clinical trial protocol and related documents.
